# Binge eating disorder and depressive symptoms among females of child-bearing age: the Korea Nurses' Health Study

**DOI:** 10.1186/s12888-018-1601-6

**Published:** 2018-01-17

**Authors:** O. Kim, M. S. Kim, J. Kim, J. E. Lee, H. Jung

**Affiliations:** 1 Korean Nurses Association, Seoul, Republic of Korea; 2 College of Nursing, Ewha Womans University, Seoul, Republic of Korea; 3 Jeju Institute of Public Health and Health Policy, Jeju, Republic of Korea; 4 Department of Nursing, Dongeui University, Busan, Republic of Korea; 5 Department of Food and Nutrition, Seoul National University, Seoul, Republic of Korea; 6 College of Nursing, Konyang University, Daejeon, Republic of Korea

**Keywords:** Binge eating disorder, Depression, DSM, Korea nurses’ health study, Nursing, Public health, South Korea

## Abstract

**Background:**

Most studies regarding the relationship between binge eating disorder (BED) and depression have targeted obese populations. However, nurses, particularly female nurses, are one of the vocations that face these issues due to various reasons including high stress and shift work. This study investigated the prevalence of BED and the correlation between BED and severity of self-reported depressive symptoms among female nurses in South Korea.

**Methods:**

Participants were 7,267 female nurses, of which 502 had symptoms of BED. Using the propensity score matching (PSM) technique, 502 nurses with BED and 502 without BED were included in the analyses. Data were analyzed using descriptive statistics, Spearman’s correlation, and multivariable ordinal logistic regression analysis.

**Results:**

The proportion of binge eating disorder was 6.90% among the nurses, and 81.3% of nurses displayed some levels of depressive symptoms. Multivariable ordinal logistic regression analysis revealed that age (40 years old and older), alcohol consumption (frequent drinkers), self-rated health, sleep problems, and stress were associated with self-reported depression symptoms. Overall, after adjusting for confounders, nurses with BED had 1.80 times the risk (95% CI = [1.41–2.30]; *p*-value < 0.001) of experiencing a greater severity of self-reported depression symptoms.

**Conclusions:**

Korean female nurse showed a higher prevalence of both binge eating disorder and depressive symptoms, and the association between the two factors was proven in the study. Therefore, hospital management and health policy makers should be alarmed and agreed on both examining nurses on such problems and providing organized and systematic assistance.

## Background

Binge eating disorder (BED) is characterized by recurring eating behaviors wherein a significantly large amount of food is consumed when an individual experiences a lack of control [1]. BED has the highest prevalence rate among all of the eating disorders [2], and this differs according to the characteristics of the subjects and the diagnostic standards. For instance, the lifetime prevalence of BED by the age of 20 years was reported as 3.0% in a study targeting a community sample of 496 female adolescents [3]. In another study, the lifetime prevalence in non-clinical samples was 2.0% for men and 3.5% for women [4]. The clinical samples consisting of obese patients seeking treatment for weight loss had a lifetime prevalence ranging from 5% to 30% [5].

BED has mostly been related to obesity or depression, and recent studies have shown comorbidity with gender (female), age (young), and high level of anxiety and stress [2]. Depression is both a risk factor of BED as well as a potential cause [5]; it is a disorder that has a high prevalence rate and is a significant health issue [6]. Women in particular have a high prevalence of depression; the prevalence rate of Koreans in 2014 was 6.7% (9.1% for women and 4.2% for men) according to the research by Korea Centers for Disease Control and Prevention [7]. According to a systematic literature review, 10 out of 14 studies showed a significant relationship between BED and depression [5]. Female BED patients showed a higher rate of loss of control than male patients [8] and reported higher levels of depression when compared to the general population [9]. Until now, however, most studies regarding the relationship between BED and depression have targeted obese populations. Therefore, it is necessary to study the nature of that association within different groups and occupations.

Among various occupational groups, nurses’ shift-works and high work stress were proven to be associated with binge eating and other abnormal eating behaviors [10]. In addition, nurses can be assumed as a high-risk group of depression as they undergo a high level of stress [11]. However, there has been a lack of research proving the association between BED and depression among nurses. Although depression has recently arisen as a major mental health problem for nurses [11], limited research was done confirming the influence of eating disorders such as binge eating on depression. This research aims to establish the association between BED and depressive symptoms among nurses by analyzing the data collected from the Korean Nurses’ Health Study, which is a large-scale prospective cohort research targeting female nurses.

## Methods

### Study design and participants

The target population was 7,267 female Registered Nurses (RNs) aged 20 to 45 years, who were in their childbearing years. Nurses with either psychiatric comorbidities or history of using psychiatric drugs were excluded. These nurses have either been medically diagnosed with depression or have taken any psychiatric drugs including Selective Serotonin Reuptake Inhibitator (SSIR) type antidepressants, other antidepressants, or Minor tranquilizers. The data was collected from July of 2013 to September of 2015 via online survey through voluntary participation. Participants had completed the first and second surveys as part of the Korea Nurses’ Health Study [12]. The Korea Nurses’ Health Study is a grand-scale research that the Korean Nurses Association and Korea Centers of Disease Control and Prevention jointly conducted using a similar survey protocol and format of the Nurses’ Health Study 3 (NHS 3) [13] of the United States. The first baseline study was conducted for 3 years, from March 2013 to December 2015. The KNHS consisted of six modules including two special modules asking about one’s early stages of pregnancy and postpartum experiences with consent in case the participants got pregnant or gave birth. This study utilized the data from the first phase of the KNHS. The second phase of the KNHS has been underway since 2016 and is scheduled to be completed in 2018. The first survey, which served as baseline data, was closed while the other surveys are still open.

### Measures

All the survey questionnaires used in this study were translated and back-translated into Korean, modified, and redeemed to suit the Korean context after discussions with an advisory committee of multidisciplinary experts. In this research, we measured whether one has binge eating disorder with 10 questions of self-report measures using the diagnostic criteria (definition of binge eating episode) of Diagnostic and Statistical Manual of Mental Disorders, fifth edition (DSM-5) [1]. The last question was a negative-form question related to inappropriate compensatory behaviors and was converted into positive-form for this research. For each question, participants could answer with either “yes” or “no,” and the BED criteria of DSM-5 was met if she answered “yes” for both of the questions: 1) did you eat an amount of food that is definitely larger than most people would eat in a similar period of time under similar circumstances in a discrete period of time? and 2) did you feel a sense of lack of control over eating? Once a participant responded “yes” to both of these questions, then five additional questionnaires were asked. These questions were: a) did you eat much more rapidly than normal? b) did you eat until feeling uncomfortably full?, c) did you eat large amounts of food when not feeling physically hungry?, d) did you eat alone because of feeling embarrassed by how much you eat?, and e) did you feel disgusted with yourself, depressed, or very guilty after overeating? Participants who answered “yes” to at least 3 of the above 5 questions and “yes” to ‘did you feel often upset both during and after the binge eating?’ and ‘did you experience binge eating episodes at least once a week over the past 3 months?’ were categorized as experiencing binge eating disorder. Additionally, we excluded participants who answered “yes” to a question regarding inappropriate compensatory behaviors like laxatives, diuretics, and self-induced vomiting.

We measured the degree of self-reported depressive symptoms using the Patient Health Questionnaire (PHQ-9), which is a self-report instrument with 9 questions. The possible score range is 0–27, divided into 0–4, 5–9, 10–14, 15–19, and above 20, representing minimal, mild, moderate, moderately severe, and severe depressive symptoms respectively. The higher the score, the severer one’s depressive symptom [14]. The Cronbach’s alpha value in our study was 0.90, and that of the original research by Arroll et al. was 0.87 [15].

In order to find the association between BED and depressive symptoms, this research considered potential confounders through literature reviews. Confounders included a) age [11], level of education [16], annual income [11], and marital status [16] as socio-demographic factors, b) alcohol consumption [17], body mass index (BMI) [11], self-rated health [18], sleep [19], stress [20] as health-behavior factors and c) shift work as a work-related factor [11].

Stress, a control variable in this study, was measured using the Perceived Stress Scale-4 (PSS-4) [21], which consists of 4 questions regarding the frequency of emotions and thoughts during the last 30 days. The possible score range is 0–16; the higher the score, the greater the degree of stress. For sleep, another control variable, we used the Jenkins Sleep Questionnaire [22], which consists of 4 questions about the amount of sleep during the last 30 days, with possible score range of 0–20; a high score implies a sleep disorder. We measured self-rated health using one question asking if a participant think of herself as healthy in three categories, which are good, fair, and poor.

### Statistical analysis

We analyzed the data using the Statistical Package for Social Sciences (SPSS) Version 23 (SPSS Inc., Chicago, IL, USA). We calculated frequency and percentage as parts of descriptive statistics and the Spearman’s correlation to identify the associations between variables. Since the data were extremely skewed with 6.9% of nurses having symptoms of binge eating, we used propensity score matching (PSM) to match the BED group and the non-BED group. This was done through estimating an average treatment effect from observational data using the following selected potential predictive variables based on prior studies: age, marital status, BMI, self-rated health, alcohol consumption, stress, sleep, and shift work. Smoking was considered as a potential predictive variable; however, almost all of the study participants were non-smokers, and we excluded it as a confounder from the analyses. After matching using the PSM, the sample size was reduced from 7,267 to 1,004 with an evenly distributed sample size of 502. The PSM technique helps to reduce the bias due to distorted samples between the group with BED and the group without it. Finally, we performed multivariable ordinal logistic regression analysis to see the relationship between BED and depressive symptoms and showed the result using odds ratios (OR) and 95% confidence intervals (CIs). The threshold for statistical significance for this study was *p* < 0.05.

## Results

### Participant characteristics

Out of the 7,267 nurses, 6.9% (*n* = 502) nurses had symptoms for BED. Distributions of key variables with and without BED as well as the differences between the two groups by key variables are presented in Table 1. According to the descriptive analysis, 81.3% of nurses displayed some levels of depressive symptoms (from mild to severe), and majority of them were younger than 30 years old (42.9%) or in the ages between 30 and 39 (45.5%). Majority of the study participants had completed either a 3-year college (51.7%) or 4-year university (42.9%), had earned either annual income in ranges of $30,000–39,999 (42.0%) or $40,000–49.999 (37.8%), and were single (76.1%). With regard to health related characteristics and shift work, majority of the nurses consumed alcohol occasionally (70.2%), had a normal BMI (61.8%), rated their health as fair (50.7%), expressed some levels of sleep problems (14.9% with the least sleep problems) and stress (26.1% with the lowest level of stress), and worked shifts (77.9%).

**Table 1:** Descriptive characteristics according to BED after matching groups using the PSM

	All (*N* = 1,004)		No (*N* = 502)		Yes (*N* = 502)		
	n	%	n	%	n	%	P
Depressive symptom							
Normal	188	18.7	95	18.9	93	18.5	0.882
Mild	386	38.4	194	38.6	192	38.2	
Moderate	23.1	23.1	116	23.1	115	22.9	
Moderately severe	140	13.9	65	12.9	75	14.9	
Severe	59	5.9	32	6.5	27	5.5	
Age (in years)							
≤ 29	431	42.9	219	43.6	212	42.2	0.895
30–39	457	45.5	225	44.8	232	46.2	
≥ 40	116	11.6	58	11.6	58	11.6	
Level of education							
Masters or higher	54	5.4	26	5.2	28	5.6	0.562
3-year college	519	51.7	268	53.4	251	50.0	
4-year university	431	42.9	208	41.4	223	44.4	
Annual income							
< $20,000	50	5.1	26	5.2	24	4.8	0.640
$20,000–$29,999	130	12.9	58	11.6	72	14.3	
$30,000–$39,999	422	42.0	208	41.4	214	42.6	
$40,000–$49,999	380	37.8	198	39.4	182	36.3	
≥ $50,000	22	2.2	12	2.4	10	2.0	
Marital status							
Single	764	76.1	394	78.5	370	73.7	0.076
Married	240	23.9	108	21.5	132	26.3	
Alcohol consumption							
Never	161	16.1	71	14.2	90	17.9	0.211
Occasionally	705	70.2	364	72.5	341	67.9	
Frequently	138	13.7	67	13.3	71	14.2	
BMI (in kg/m^2^)^*^							
Normal (18.5–22.9)	621	61.8	308	61.4	313	62.3	0.000
Underweight (< 18.5)	114	11.4	81	16.1	33	6.6	
Overweight (≥23)	269	26.8	113	22.5	156	31.1	
Self-rated health							
Poor	188	18.7	94	18.7	94	18.7	0.937
Fair	509	50.7	257	51.2	252	50.2	
Good	307	30.6	151	30.1	156	31.1	
Sleep (quartile)							
1 (least sleep problem)	150	14.9	74	14.7	76	15.1	0.946
2	263	26.2	128	25.5	135	26.9	
3	252	25.1	127	25.3	125	24.9	
4	339	33.8	173	34.5	166	33.1	
Stress (quartile)							
1 (least stressed)	262	26.1	139	27.7	123	24.5	0.584
2	301	30.0	146	29.1	155	30.9	
3	256	25.5	122	24.3	134	26.7	
4	185	18.4	95	18.9	90	17.9	
Shift work							
No	222	22.1	106	21.1	116	23.1	0.447
Yes	782	77.9	396	78.9	386	76.9	

*Note*: Occasionally: sometimes in a month; Frequently: sometimes in a week; BMI: body mass index

^*^BMI categories are according to the World Health Organization Asia Pacific Region and the Korean Society for the Study of Obesity

### BED and severity of self-reported depressive symptoms

Multicollinearity was examined with no variance inflation factor (VIF) values over 10 [23]. Spearman’s correlation analyses showed that all variables showed moderate correlation below 0.6 (Table 2), which justified proceeding to conduct multivariable ordinal logistic regression [24]. Table 3 shows the results of the multivariable ordinal logistic regression analysis. In model 2, socio-demographic characteristics, such as age, level of education, annual income, and marital status, were controlled; health behaviors including alcohol consumption, BMI, self-rated health, sleep, and stress were included as control variables in Model 3. In the final model (Model 4), shift work was also included. Out of the control variables, age (40 years old and older), alcohol consumption (frequent drinkers), self-rated health, sleep problems, and stress were associated with depressive symptoms in the final model. Overall, after adjusting for confounders, nurses with binge eating disorders had 1.80 times the risk of experiencing a greater severity of self-reported depressive symptoms when compared to those without BED. Table 3 shows that as we add more confounders, both the Nagelkerke R-square values and the risk of nurses with binge eating disorders to experience a greater severity of depressive symptoms increase.

**Table 2:** Correlation coefficients for variables

		1	2	3	4	5	6	7	8	9	10	11	12
1	Depressive symptoms	1											
2	BED	.008	1										
3	Age	.012	−.177^**^	1									
4	Level of education	.025	.014	.131^**^	1								
5	Annual income	.040	−.050	.430^**^	.112^**^	1							
6	Marital status	.056	−.209^**^	.542^**^	.078^*^	−.241^**^	1						
7	Alcohol consumption	−.028	.001	−.050	−.051	.005	−.033	1					
8	BMI	.026	.001	.157^**^	−.037	.004	.174^**^	.005	1				
9	Self-rated health	.008	−.403^**^	.006	.020	−.015	.069^*^	.015	−.051	1			
10	Sleep	−.017	.513^**^	−.113^**^	.000	.053	−.182^**^	.035	.021	−.332^**^	1		
11	Stress	.019	.392^**^	−.118^**^	−.001	.108^**^	−.113^**^	−.004	−.056	−.253^**^	.189^**^	1	
12	Shift work	−.024	.171^**^	−.269^**^	−.066^*^	.026	−.303^**^	.083^**^	−.019	−.099^**^	.227^**^	.079^*^	1

*Note*: BMI: body mass index

^*^p < .05, ^**^p < .01, ^***^p < .001

**Table 3:** Result of the multivariate ordinal logistic regression analysis

	Model 1		Model 2		Model 3		Model 4	
Variables	*OR*	95% *CI*	*OR*	95% *CI*	*OR*	95% *CI*	*OR*	95% *CI*
BED								
No	Ref.		Ref.		Ref.		Ref.	
Yes	1.456**	1.163–1.823	1.537**	1.223–1.931	1.795**	1.410–2.286	1.800**	1.414–2.292
Age (in years)								
≤ 29			Ref.		Ref.		Ref.	
30–39			0.904	.685–1.193	0.780	0.581–1.047	0.785	0.585–1.055
≥ 40			0.308**	0.176–0.536	0.359**	0.195–0.659	0.372**	0.200–0.690
Level of education								
3-year college			Ref.		Ref.		Ref.	
4-year college			1.096	0.857–1.401	1.122	0.866–1.454	1.130	0.872–1.464
Master or higher			1.251	0.686–2.279	1.321	0.700–2.496	1.320	0.699–2.494
Annual income								
< $20,000			Ref.		Ref.		Ref.	
$20,000–29,999			1.140	0.618–2.103	0.798	0.412–1.544	0.800	0.413–1.549
$30,000–39,999			1.378	0.775–2.448	0.913	0.491–1.699	0.916	0.492–1.706
$40,000–49,999			1.332	0.733–2.419	0.860	0.451–1.639	0.873	0.457–1.666
≥ $50,000			0.965	0.361–2.577	0.546	0.194–1.538	0.556	0.197–1.569
Marital status								
Single			Ref.		Ref.		Ref.	
Married			1.691**	1.217–2.351	1.393	0.983–1.974	1.371	0.964–1.951
Alcohol consumption								
Never					Ref.		Ref.	
Occasionally					0.759	0.543–1.062	0.759	0.543–1.062
Frequently					0.572*	0.365–0.897	0.565*	0.360–0.887
BMI, kg/m^2^								
Normal (18.5–22.9)					Ref.		Ref.	
Underweight (< 18.5)					1.356	0.910–2.019	1.354	0.909–2.018
Overweight(≥ 23)					1.087	0.821–1.440	1.084	0.818–1.435
Self-rated health								
Poor					Ref.		Ref.	
Fair					0.457**	0.327–0.639	0.454**	0.325–0.635
Good					0.317**	0.215–0.468	0.317**	0.214–0.467
Sleep (quartile)								
1 (least problem)					Ref.		Ref.	
2					2.821**	1.852–4.295	2.783**	1.823–4.246
3					5.439**	3.511–8.425	5.341**	3.438–8.299
4					12.494**	7.991–19.535	12.272**	7.823–19.253
Stress (quartile)								
1 (least stress)					Ref.		Ref.	
2					2.094**	1.493–2.937	2.092**	1.492–2.934
3					3.671**	2.583–5.217	3.650**	2.568–5.190
4					7.346**	4.907–10.998	7.310**	4.882–10.945
Shift work								
No							Ref.	
Yes							0.903	0.656–1.243
Nagelkerke R^2^	0.011^**^		0.092^***^		0.421^***^		0.421^***^	
x^2^/df	10.758/1		91.507/10		507.345/22		507.717/23	

*Note*: *OR*: odds ratio; 95% *CI*: 95% confidence interval; Ref: reference group; Occasionally: sometimes in a month; Frequently: sometimes in a week; BMI: body mass index

^*^p < .05, ^**^p < .01, ^***^p < .001

## Discussion

First, we confirmed high prevalence rates in both BED and self-reported depressive symptoms among Korean female nurses. While the BED prevalence rate in a prior research targeting a community sample by using the same DSM-5 criteria was 3.0% [3], 6.9% of our research targets met the BED criteria. Besides this, we found that the prevalence rate in our research targeting female nurses was higher even when compared to the BED prevalence rate of 3.53% from a study done on female college students in Wuhan, China [25]. Similarly, the prevalence rate of BED from a phone interviewed study of Canadian women was 3.8% [26], and the rate from an Australian adolescent cohort study was 5.6% [27]. In the current research, the high BED prevalence rate supports that the female nurses are a BED high-risk group, corroborated by a previous research [10].

The prevalence of depressive symptoms among Korean nurses in the current research was 81.2% (38.4% with mild, 23.1% with moderate, 13.9% with moderately severe and 5.9% with severe); we could confirm that the level of depressive symptoms among Korean nurses was considerably high. Prior studies showed lower prevalence rates [28, 29]. A cross-sectional research conducted on 441 Korean nurses using the South Korean version of the Center for Epidemiologic Studies rating scale for Depression (CESD) showed a prevalence rate of 38% [28]. Another prior study done on 1,592 nurses of hospitals in Liaoning, China, using the PHQ-P measurement, showed a prevalence rate 61.7% [29].

Using the BED criteria of the DSM-5 and PHQ-9, this study confirmed the association between BED and self-reported depressive symptoms among female nurses. In our research, the results from the multivariable ordinal logistic regression showed that the nurses with BED were 1.80 times more likely to have self-reported depressive symptoms compared with the nurses without BED controlling the potential confounders. Previous researches have also shown that BED was significantly related to depression including a study by Borges [30] targeting Brazilian women who participated in a weight loss program and Celic et al. [31] showing the connection between BED and the level of depression within a type 2 diabetes mellitus (T2DM) patient group. Other studies also confirmed the association between the two variables including a study revealing the high correlation between BED and depression in general adult population [32] and another study on young women in the United States [33]. Overall, we can say that the result of this research was a reconfirmation of the fact that BED is a major influential factor of depressive symptoms.

Although female nurses are rationally a group to be studied on the association between BED and depression, limited studies have examined the association among female nurses. We have seen from our own data of Korea Nurses’ Health Study that Korean female nurses show high prevalence of self-reported depressive symptoms, but majority of them do not seek medical help [11]. This alarmed us and made us to further investigate the associations between depressive symptoms and other health issues among the population. Our findings might not bring any brilliantly new clinical findings to the current understanding of binge eating and depression. However, we strongly believe that it is meaningful to investigate the association between the two factors among female nurses since they show high prevalence in both BED and depressive symptoms and are not investigated enough compare to other groups.

This research has both strengths and limitations. Our research outcome is as unique as a pioneer research that found a relationship between BED and depressive symptoms among a specific occupation group. It is also quite significant that we confirmed the BED prevalence rate, while excluding the comorbidity of night eating syndrome; we also saw a meaningful association between self-reported BED symptoms and depressive symptoms, especially on a non-clinical population. Despite these significant advantages, there are some limitations in this research. First, while we controlled certain related variables to find the association between BED and depressive symptoms, we failed to control other potential confounders, such as a dissatisfaction about one’s body image and other work or family related factors, which should be considered in future researches. Second, we used only the DSM-5 criteria to diagnose BED. Using a broader measurement, such as the DSM-5 criteria could have influenced the high prevalence rate of BED in our study. For further studies, we suggest more conservative measurement of BED by combining self-report measures to identify BED symptoms with a 2-stage screening processes either including expert interviews or additional surveys. Lastly, our research only included Korean female RNs aged 20 to 45. Findings may not be generalized beyond this specific population. Therefore more research in this area needs to be conducted on different study subjects including various occupations and ethnicities.

## Conclusions

This research aimed to find the relationship between BED and depressive symptoms among Korean female nurses, and we were able to confirm a high correlation between the two variables as a pioneer study. Currently, depression is a major mental and psychological health problem in the Korean society**,** and eating disorders including BED are not gaining enough attention in Korea. Based on the findings of this study, hospital management and health policy makers should be alarmed and agreed on both examining nurses on such problems and providing organized and systematic assistance.

## Abbreviation

BED: Binge eating disorder
DSM-5: Diagnostic and Statistical Manual of Mental Disorders, fifth edition
KNHS: Korea Nurses’ Health Study
NHS: Nurses’ Health Study
PHQ-9: Patient Health Questionnaire-9
PSM: Propensity Score Matching
